# Effects of *APOE2* and *APOE4* on brain microstructure in older adults: modification by age, sex, and cognitive status

**DOI:** 10.1186/s13195-023-01380-w

**Published:** 2024-01-11

**Authors:** Emilie T. Reas, Curtis Triebswetter, Sarah J. Banks, Linda K. McEvoy

**Affiliations:** 1 Department of Neurosciences, University of California, San Diego, Mail Code 0841, UCSD,9500 Gilman Dr., La Jolla, San Diego, CA 92093-0841 USA; 2 Herbert Wertheim School of Public Health and Human Longevity Science, University of California, San Diego, USA; 3 Kaiser Permanente Washington Health Research Institute, Seattle, WA USA

**Keywords:** APOE, Diffusion MRI, Brain microstructure, Genetics, Brain aging, Neuroimaging, Sex differences

## Abstract

**Background:**

*APOE4* is the strongest genetic risk factor for sporadic Alzheimer’s disease (AD), whereas *APOE2* confers protection. However, effects of *APOE* on neurodegeneration in cognitively intact individuals, and how these associations evolve with cognitive decline, are unclear. Furthermore, few studies have evaluated whether effects of *APOE* on neurodegenerative changes are modified by other AD key risk factors including age and sex.

**Methods:**

Participants included older adults (57% women; 77 ± 7 years) from the Rancho Bernardo Study of Health Aging and the University of California San Diego Alzheimer’s Disease Research Center, including 192 cognitively normal (CN) individuals and 33 with mild cognitive impairment. Participants underwent diffusion MRI, and multicompartment restriction spectrum imaging (RSI) metrics were computed in white matter, gray matter, and subcortical regions of interest. Participants were classified as *APOE4* carriers, *APOE2* carriers, and *APOE3* homozygotes. Analysis of covariance among CN (adjusting for age, sex, and scanner) assessed differences in brain microstructure by *APOE*, as well as interactions between *APOE* and sex. Analyses across all participants examined interactions between *APOE4* and cognitive status. Linear regressions assessed *APOE* by age interactions.

**Results:**

Among CN, *APOE4* carriers showed lower entorhinal cortex neurite density than non-carriers, whereas *APOE2* carriers showed lower cingulum neurite density than non-carriers. Differences in entorhinal microstructure by *APOE4* and in entorhinal and cingulum microstructure by *APOE2* were present for women only. Age correlated with lower entorhinal restricted isotropic diffusion among *APOE4* non-carriers, whereas age correlated with lower putamen restricted isotropic diffusion among *APOE4* carriers. Differences in microstructure between cognitively normal and impaired participants were stronger for *APOE4*-carriers in medial temporal regions, thalamus, and global gray matter, but stronger for non-carriers in caudate.

**Conclusions:**

The entorhinal cortex may be an early target of neurodegenerative changes associated with *APOE4* in presymptomatic individuals, whereas *APOE2* may support beneficial white matter and entorhinal microstructure, with potential sex differences that warrant further investigation. *APOE* modifies microstructural patterns associated with aging and cognitive impairment, which may advance the development of biomarkers to distinguish microstructural changes characteristic of normal brain aging, *APOE*-dependent pathways, and non-AD etiologies.

**Supplementary Information:**

The online version contains supplementary material available at 10.1186/s13195-023-01380-w.

## Background

Apolipoprotein E (*APOE)* is a polymorphic lipoprotein with three predominant isoforms that modify risk for sporadic Alzheimer’s disease (AD). Compared to ε3 homozygotes, individuals with one or two ε4 alleles harbor approximately 3 and 15 times increased risk, respectively, whereas carriers of the protective ε2 allele have an estimated 1.7 times decreased risk [1]. *APOE* is multifunctional, with roles in lipid and glucose metabolism, neuroinflammation, amyloid and tau accumulation, and blood-brain barrier dysfunction [2]. However, the downstream regulation of neurodegeneration or neuroprotection by *APOE*-dependent pathways remains elusive, due in part to the challenges of in vivo approaches that are typically limited in their measurement of subtle cytoarchitectural changes. Improved characterization of *APOE* effects on brain microstructure in non-demented older adults may be dually instrumental in optimizing preclinical AD biomarkers and therapeutic strategies targeting *APOE*-dependent pathways.

Diffusion MRI studies have identified microstructural white matter (WM) abnormalities in cognitively normal older *APOE4* carriers, including increased mean diffusivity and reduced fractional anisotropy or restricted diffusion [3–5], whereas others observed null [6, 7] or inconsistent [8] effects. Prior discrepancies may be attributable to cohort differences in factors such as age range, health, or socioeconomic status, to methodological differences in diffusion MRI techniques, or inclusion of *APOE2* carriers in the reference group. Whereas previous studies have predominantly focused on WM integrity, more recent investigations have interrogated cortical or subcortical gray matter with inconclusive findings [9, 10]. Further investigation of *APOE* effects on regions that are early targets of AD pathology, including the entorhinal cortex and hippocampus, is warranted.

Uncertainty persists regarding the potential neuroprotective effects of *APOE2* on brain aging, due largely to its low population prevalence of only 10%, making it understudied relative to *APOE4*. Although lower amyloid burden has been observed among *APOE2* carriers, differences in tau or atrophy have been inconclusive [11, 12], with some reports of slower hippocampal atrophy among *APOE2* carriers [13]. The few diffusion MRI studies evaluating *APOE2* have similarly generated mixed findings, with one study observing higher WM fractional anisotropy among *APOE2* carriers [14], yet others reporting null effects [7, 15]. Thus, despite the established protective effect of *APOE2* against AD, it remains unclear whether higher brain reserve in the form of preserved cytoarchitectural integrity, underlies this resistance to AD.

*APOE* is differentially associated with AD risk across populations [16], such that key AD risk factors, including older age and female sex, may obscure associations of *APOE* with neurodegenerative trajectories. Consistent with accelerating *APOE4*-dependent risk for AD and cognitive decline with older age [17], some studies have reported stronger effects of *APOE4* on WM microstructure with age [3, 15, 18], whereas others observed no such interaction [8]. Although sex differences in effects of *APOE* on microstructure have been scarcely examined, studies reporting sex-specific associations of *APOE4* with WM integrity [19] and stronger *APOE*-related differences in brain metabolism and cortical atrophy for women than men [20, 21] provide grounds for deeper exploration of sex differences in underlying cytoarchitecture.

Considering the prolonged preclinical period preceding AD onset, identifying the earliest *APOE*-dependent neurodegenerative changes may provide a window of opportunity for timely detection at critical points along the disease course, warranting focus on asymptomatic individuals. However, given the heterogeneous nature of mild cognitive impairment (MCI), characterizing neurodegenerative patterns that manifest with AD-specific risk may help to develop biomarkers that aid in more accurate differential diagnosis. While prior diffusion MRI studies have focused on cognitively normal individuals or those with AD dementia, limited evidence suggests that *APOE4* may drive accelerated hippocampal atrophy and network disruption with MCI [22], though others report no modifying effect of *APOE4* on WM microstructure in MCI [23]. Thus, further research interrogating differential markers of cytoarchitectural injury according to *APOE* across the preclinical to prodromal AD continuum may help to inform the probable etiology of nascent cognitive decline.

Thus, further research is needed to characterize *APOE*-related patterns of microstructural brain aging while considering key AD risk modifiers, to improve precision diagnostic approaches for targeted early disease detection. In this study, we employed restriction spectrum imaging (RSI) [24], a multicompartment diffusion MRI model that offers more comprehensive characterization of tissue cytoarchitecture than morphometric MRI or diffusion tensor imaging (DTI), to examine effects of *APOE2* and *APOE4* on brain microstructure in older adults across the continuum from cognitively normal to mildly impaired. Building upon our prior work demonstrating excellent sensitivity of RSI metrics to microstructural brain injury in MCI [25, 26], and to more subtle cytoarchitectural changes that manifest with normal aging [27], herein we further probe effects of *APOE* on brain microstructure and their modification by age and sex.

## Methods

### Participants

Eligible participants included predominantly non-Hispanic White community-dwelling participants of the Rancho Bernardo Study (RBS) of Healthy Aging and the UC San Diego Shiley-Marcos Alzheimer’s Disease Research Center (ADRC) longitudinal study who completed a diffusion MRI scan and had available genetic data. MRI data were acquired for RBS participants between 2014 and 2016 and for ADRC participants from 2013 to 2022. ADRC participants underwent consensus diagnosis by two senior neurologists, and those with diagnoses of cognitively normal (CN), MCI [28], or mild AD [29], were eligible for study inclusion. RBS participants did not undergo clinical evaluation but completed the Modified Mini-Mental State Exam (3MS), a cognitive screening tool for the assessment of dementia. Cognitively impaired (CI) individuals included ADRC participants with diagnoses of MCI or mild AD and RBS participants with a 3MS score < 78 [30]. Exclusion criteria included history of head injury, stroke, dementia, neurological disease, treatment for an alcohol use disorder, or safety contraindication for MRI. After excluding six participants due to poor MRI data quality and three participants with *APOE2/4* genotype due to conflicting risk effects of the ε2 and ε4 alleles, the final sample included 225 participants (139 RBS, 86 ADRC; 192 CN, 33 CI; 57% women; age at MRI: mean ± SD 76.5 ± 7.0, range 56–97 years).

### Standard protocol approvals and participant consents

Study procedures were approved by the University of California, San Diego Human Research Protections Program Board and participants provided informed written consent prior to participation.

### Demographic and health assessment

Education level was acquired at enrollment and converted to years of education. History of medical conditions was obtained from standard questionnaires. Blood pressure was measured in seated, resting participants. Participant height and weight were measured to compute body mass index (BMI, kg/m^2^).

### APOE genotyping

For RBS participants, DNA was extracted by Sequana Therapeutics (La Jolla, CA) using standard techniques (Puregene; Gentra, Minneapolis, MN) and genotyping was conducted by Diagnomics, Inc. Genotyping for ADRC participants used a commercially available Illumina BeadChip array. Participants were classified as *APOE2/3*, *APOE4* carrier (*APOE3/4* or *APOE4/4*), or *APOE3/3* (there were no *APOE2* homozygotes and *APOE2/4* participants were excluded from analysis). One participant with an inconclusive genotype of *APOE2/3* versus *APOE3/3* was excluded from *APOE2* analyses.

### Amyloid and tau measurement

A subset of ADRC participants (*N* = 70) underwent lumber puncture with standardized procedures, preanalytical preparation, and storage of cerebrospinal fluid in accordance with the recommended best practices [31]. Briefly, lumbar puncture was conducted early in the morning after overnight fasting to collect 15–25 mL cerebrospinal fluid. Samples were processed, aliquoted into 500 μL fractions in polypropylene microtubes, snap-frozen, and stored at -80 °C until assayed. Samples were analyzed with the automated Lumipulse platform using assays developed with established monoclonal antibodies (Fujirebio Inc.) to measure beta-amyloid (Aβ)-42 and 40, total tau (t-tau), and phosphorylated tau (p-tau). T-tau/Aβ42 > 0.54 was considered positive for AD pathology [32]. Because only a subset of participants (31% of the full sample) underwent lumbar puncture, cerebrospinal fluid measures are reported for AD biomarker characterization and were not incorporated into analyses.

### Imaging data acquisition

Imaging data were acquired on two 3.0 Tesla Discovery 750 scanners (GE Healthcare, Milwaukee, WI, USA) at the University of California, San Diego Center for Functional MRI (100% of RBS participants and 58% of ADRC participants) and the Altman Clinical and Translational Research Institute (42% of ADRC participants), using an eight-channel phased array head coil. MRI sequences common to the RBS and ADRC protocols included a three-plane localizer; a sagittal 3D fast spoiled gradient echo T_1_-weighted structural scan optimized for maximum tissue contrast (TE = 3.2 ms, TR = 8.1 ms, inversion time = 600 ms, flip angle = 8°, FOV = 256 × 256 mm, matrix = 256 × 192, slice thickness = 1.2 mm, resampled to a 1 × 1 × 1.2 mm resolution, scan time 8:27); and an axial 2D single-shot pulsed-field gradient spin-echo echo-planar diffusion-weighted sequence (45 gradient directions, b-values = 0, 500, 1500, 4000 s/mm^2^, one b = 0 volume and 15 gradient directions for each non-zero b-value; TE = 80.6 ms, TR = 7 s, FOV = 240 × 240 mm, matrix = 96 × 96, slice thickness = 2.5 mm, resampled to a 1.875 × 1.875 × 2.5 mm resolution, scan time 6:34).

### Data processing

All raw and processed MR images were visually inspected for artifacts and processed using an automated image processing pipeline that integrates FreeSurfer (http://surfer.nmr.mgh.harvard.edu) with in-house software [33]. Cortical gray matter, WM, and CSF boundaries were reconstructed from T_1_-weighted structural images using FreeSurfer (version 5.3) and subcortical regions were automatically segmented according to a subcortical atlas [34]. Using previously detailed methods [33], diffusion MRI data underwent eddy current correction in the phase-encode direction with displacements modeled as a function of spatial location, gradient orientation, and gradient strength, and correction for head motion with rigid-body registration. Spatial and intensity distortions caused by B_0_ field inhomogeneity were corrected by aligning b = 0 images acquired in opposite phase encoding direction using nonlinear registration and correcting subsequent images using the displacement volume. The b = 0 images were registered to T_1_ images using mutual information after coarse pre-alignment to atlas brains, and diffusion images were aligned with a fixed rotation and translation relative to the T_1_ image. For cortical surface-based analyses, RSI metrics were sampled with linear interpolation from 0.8 to 2.0 mm from the gray/white matter boundary and, to minimize partial volume effects, were computed using a weighted average based on the proportion of gray matter in each voxel [35]. RSI cortical surface maps were registered to common space and smoothed with a FWHM 10 mm kernel. WM fiber tracts were labeled using AtlasTrack, a WM fiber atlas based on prior probability and orientation information [36], and voxels containing primarily gray matter or CSF were excluded from WM [34]. T_1_ images were used to nonlinearly coregister brains to common space and diffusion orientation estimates were compared to atlas orientations to refine voxel-wise probabilities of belonging to a given fiber. All raw and processed structural and diffusion images underwent visual quality control. Manual editing of the cortical surface reconstruction, including adding white matter control points or removing mislabeled non-brain voxels, was conducted when applicable; the majority of scans underwent at least minimal editing.

### Computation of RSI metrics

For primary analyses, RSI measures were computed in regions of interest (averaged between left and right hemispheres), identified based upon their early involvement in AD, including the entorhinal cortex and hippocampus, which are targets of early AD neuropathology and neurodegeneration, and eight WM limbic and association fibers (cingulum, corpus callosum, fornix, inferior fronto-occipital fasciculus, inferior longitudinal fasciculus, parahippocampal cingulum, superior longitudinal fasciculus, and uncinate). Exploratory analyses also examined RSI measures in global white and cortical gray matter, across cortical gray matter surface vertices, and in four additional subcortical regions (thalamus, putamen, caudate, and amygdala). Global white and gray matter measures were calculated as the mean across all WM fibers or all cortical gray matter, respectively. Computed metrics included restricted isotropic diffusion (RI), a measure of highly restricted, non-oriented diffusion that likely corresponds with the intracellular fraction; neurite density (ND), a measure of oriented restricted diffusion that accounts for multiple diffusion orientations, presumably reflecting cellular processes such as axons, dendrites, or glial processes; hindered isotropic diffusion (HI), a measure of non-restricted diffusion that is hindered by cellular barriers and consistent with diffusion within large cell bodies or the extracellular space; and isotropic free water (IF), a measure of cerebrospinal fluid (Additional file 1: Table S1) [24, 27]. All measures were computed in cortical and subcortical gray matter, while HI was not examined in WM fibers due its poor representation in WM [24].

### Statistical analysis

Differences in demographic and health factors by *APOE* genotype were examined using analyses of variance for continuous variables or chi-squared tests for categorical variables. BMI was adjusted for sex.

Analyses of RSI measures included covariates of age, sex, and scanner. To examine effects of *APOE4* on brain microstructure in asymptomatic individuals, analyses of covariance (ANCOVA) were conducted among CN with *APOE4* (carrier versus non-carrier) as the independent variable and RSI metric as the dependent variable. To evaluate effects of *APOE2*, models were repeated with *APOE4* non-carriers further classified as *APOE2/3* or *APOE3/3*.

To evaluate sex differences in associations between *APOE4* or *APOE2* and brain microstructure, models were repeated with a term for the interaction between sex and *APOE* status.

To assess modification of age-microstructure associations by *APOE*, linear regressions were conducted with factors of *APOE*, age, and the interaction between *APOE* and age. Secondary models included additional factors of age^2^ and the interaction between *APOE* and age^2^ to assess possible nonlinear age effects. Interaction models used mean-centered variables to reduce multicollinearity. For any RSI measure demonstrating significant interactions, *APOE*-stratified analyses were performed.

Finally, to probe whether *APOE4* modified effects of cognitive impairment on microstructure, ANCOVAs were conducted with factors of *APOE4* and cognitive status (CN versus CI) and their interaction. Significant interactions were followed by analysis of cognitive status stratified by *APOE4*.

Region-of-interest analyses were conducted in SPSS version 28.0 (IBM Corp, Armonk, NY, USA) and cortical surface analyses were performed in FreeSurfer version 6.0. Significance was set to *p* < 0.05. Uncorrected *p*-values are reported for transparency, but to account for multiple comparisons across eight fibers and four subcortical regions, significance was assessed using Bonferroni corrected thresholds set to *p* < 0.006 for WM fibers and *p* < 0.012 for subcortical regions. Cortical surface general linear models were corrected with the false discovery rate (FDR) method.

## Results

### Participant characteristics

Participant characteristics by *APOE4* (74 carriers, 151 non-carriers) for the full sample, and stratified by cognitive status, are shown in Additional file 1: Table S2. *APOE4* carriers (28%) were more likely to be cognitively impaired (*p* < 0.001) than non-carriers (8%) and had higher diastolic blood pressure (*p* = 0.009). Further analysis of diastolic blood pressure identified a sex by *APOE4* interaction among CN (*F*(1,182) = 5.87, *p* = 0.02), such that the difference was only present among men (*p* < 0.001) but not women (*p* = 0.31) (full sample: men *p* = 0.03, women *p* = 0.09). Participant age, sex, years of education, systolic blood pressure, BMI, or diabetes did not differ by *APOE4* (*p* > 0.05). CI participants were more likely to be male and more highly educated than CN participants (*p* < 0.001; Additional file 1: Table S3). *APOE2/3* participants did not differ from *APOE2* non-carriers on any demographic or health factor (Table 1). The subset of 70 participants who underwent lumbar puncture included 31% CI, 47% *APOE4* carriers, and 54% women and had a mean age of 75.5 ± 5.6 years and mean time between MRI and lumbar puncture of 2.4 ± 2.2 years. *APOE4* carriers and CI participants were more likely to be positive for AD pathology (tau/Aβ42, *p* < 0.05), and to have lower Aβ42/40 and higher p-tau than *APOE4* non-carriers and CN participants (*p* < 0.01) (Additional file 1: Table S4). Participant characteristics by cohort are presented in Additional file 1: Table S5.

**Table 1:** Participant characteristics (mean ± SD or *N*(%)) by *APOE* genotype for all subjects and cognitively normal subjects

	**All (*N* = 225)**				**Cognitively normal (*N* = 191)**			
	**APOE2/3** ***N* = 22**	**APOE3/3** ***N* = 128**	**APOE4 + ** ***N* = 74**	**Group difference**	**APOE2/3** ***N* = 22**	**APOE3/3** ***N* = 116**	**APOE4 + ** ***N* = 53**	**Group difference**
Age (years)	77.4 ± 8.1	76.8 ± 7.2	75.8 ± 6.1	*F*(2,221) = 0.72,* p* = 0.49	77.4 ± 8.1	76.4 ± 7.1	75.4 ± 5.6	*F*(2,188) = 0.74,* p* = 0.48
Sex (women)	*N* = 12 (55%)	*N* = 73 (57%)	*N* = 44 (59%)	*X*^2^(2) = 0.21,* p* = 0.90	*N* = 12 (55%)	*N* = 70 (60%)	*N* = 37 (70%)	*X*^2^(2) = 2.03,* p* = 0.36
Education (years)	14.8 ± 2.1	15.5 ± 2.3	16.0 ± 2.5	*F*(2,221) = 2.15,* p* = 0.12	14.8 ± 2.1	15.3 ± 2.2	15.5 ± 2.4	*F*(2,188) = 0.71,* p* = 0.49
SBP	125.2 ± 21.8	125.3 ± 14.8	129.3 ± 17.9	*F*(2,215) = 1.38,* p* = 0.25	125.2 ± 21.8	125.5 ± 15.2	130.0 ± 18.8	*F*(2,182) = 1.29,* p* = 0.28
DBP	70.7 ± 11.7	71.5 ± 9.2	75.0 ± 9.3*	*F*(2,215) = 3.49,* p* = 0.03	70.7 ± 11.7	71.5 ± 9.1	75.4 ± 9.0*	*F*(2,182) = 3.43,* p* = 0.03
BMI	25.7 ± 3.6	25.8 ± 4.3	25.0 ± 3.6	*F*(2,217) = 0.90,* p* = 0.41	25.6 ± 3.6	25.7 ± 4.1	25.1 ± 3.7	*F*(2,184) = 0.38,* p* = 0.68
Diabetes	*N* = 4 (18%)	*N* = 19 (15%)	*N* = 9 (12%)	*X*^2^(2) = 0.59,* p* = 0.74	*N* = 4 (18%)	*N* = 16 (14%)	*N* = 6 (11%)	*X*^2^(2) = 0.63,* p* = 0.73
Cognitively impaired	0 (0%)	12 (9%)	21 (28%)	*X*^2^(2) = 17.86,* p* < 0.001				

Body mass index (BMI) is adjusted for sex. *DBP* diastolic blood pressure, *SBP* systolic blood pressure

^*^p < 0.05 versus *APOE3/3*, Bonferroni corrected for multiple comparison

### Effects of APOE4 on brain microstructure

**Fig. 1:**
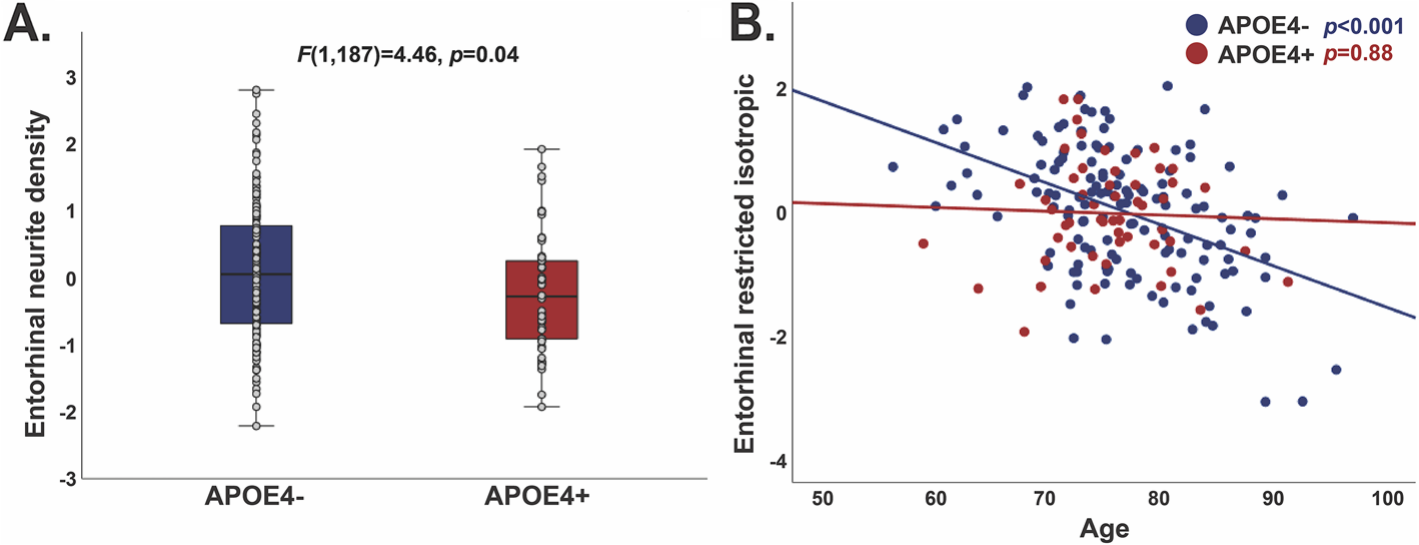
Differences in entorhinal cortex microstructure by *APOE4* among cognitively normal participants. Entorhinal cortex neurite density was lower for cognitively normal *APOE4* carriers than non-carriers (**A**). Entorhinal restricted isotropic diffusion correlated with age only for *APOE4* non-carriers (**B**). Values are residuals, adjusted for age, sex, and scanner

When brain microstructure was examined among CN according to *APOE4* status, entorhinal cortex ND was lower for *APOE4* carriers compared to non-carriers (*F*(1,187) = 4.46,* p* = 0.04; Additional file 1: Table S6; Fig. 1A). This difference was unchanged by adjustment for entorhinal cortex thickness (*F*(1,185) = 4.39,* p* = 0.04). Sex-stratified analyses revealed that entorhinal ND differed by *APOE4* only for women (*F*(1,115) = 4.90,* p* = 0.03) but not for men (*F*(1,69) = 0.25,* p* = 0.62) (Additional file 1: Figure S1A). However, sex did not interact with *APOE4* for entorhinal ND (*p* = 0.42) or any other measure. Because male *APOE4* carriers had higher diastolic blood pressure than non-carriers, analyses were further adjusted for diastolic blood pressure. Differences in entorhinal ND were moderately attenuated for the full sample (*F*(1,186) = 2.23, *p* = 0.14) and trivially changed among women (*F*(1,114) = 3.60,* p* = 0.06), for whom blood pressure did not differ by *APOE4*. Age interacted with *APOE4* for entorhinal cortex RI (*p* = 0.02), with correlations between older age and lower RI for *APOE4* non-carriers (*p* < 0.001) but not carriers (*p* = 0.88) (Fig. 1B). This interaction remained after adjustment for entorhinal cortex thickness (*p* = 0.02) and diastolic blood pressure (*p* = 0.04). Exploratory analyses revealed no further differences by *APOE4* nor interactions with age or sex, across the cortical gray matter surface (*p* < 0.05 FDR corrected), in global white or cortical gray matter, or within subcortical regions (Bonferroni corrected).

### Effects of APOE2 on brain microstructure

**Fig. 2:**
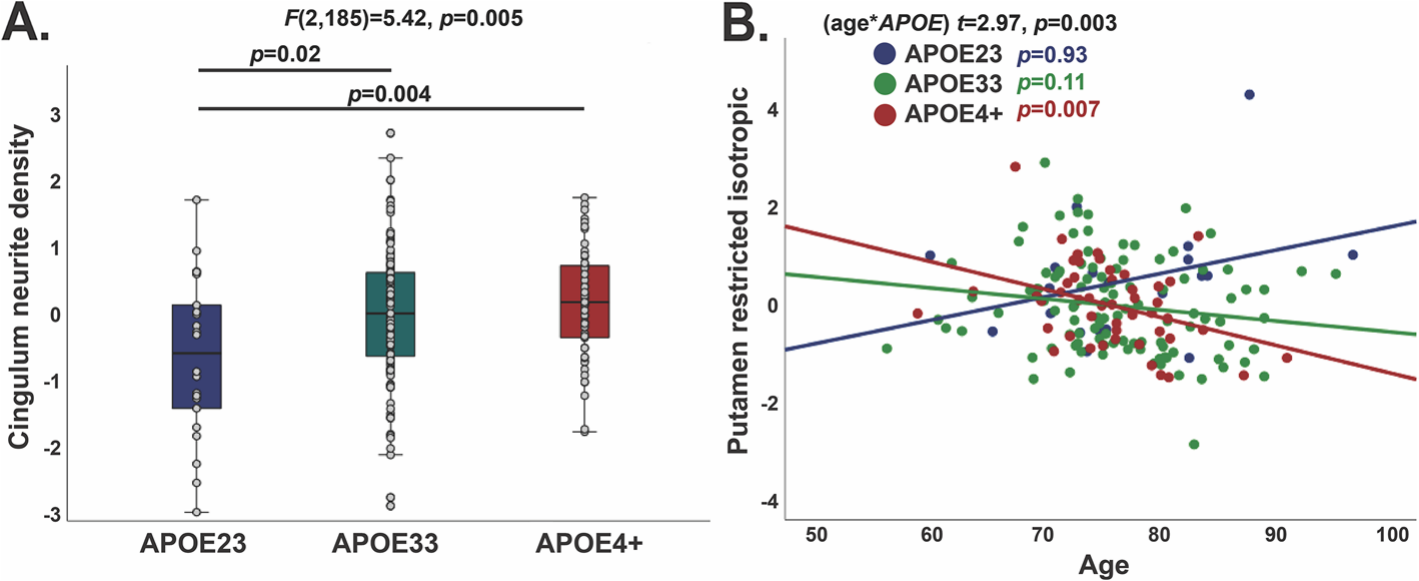
Effects of *APOE2* on brain microstructure among cognitively normal participants. Cingulum neurite density was lower for cognitively normal *APOE2/3* than for *APOE2* non-carriers (**A**). *APOE* interacted with age on putamen restricted isotropic diffusion, with correlations present for *APOE4* carriers only (**B**). Values are residuals, adjusted for age, sex, and scanner

To probe differences by *APOE2*, *APOE4* non-carriers were further classifed as *APOE2/3* (there were no *APOE2* homozogotes) and *APOE3* homozygotes. Across all CN participants, cingulum ND differed by *APOE2* (*F*(2,185) = 5.42,* p* = 0.005), with lower ND for *APOE2/3* than *APOE3/3* and *APOE4* carriers (Additional file 1: Table S7; Fig. 2A). In sex-stratified analyses, this difference was present for women (*F*(2,114) = 6.33,* p* = 0.002) but not for men (*F*(2,67) = 0.70,* p* = 0.50) (Additional file 1: Figure S1C). Similarly, there was an effect of *APOE2* on entorhinal HI for women (*F*(2,114) = 4.43,* p* = 0.01) but not men (*F*(2,67) = 0.10,* p* = 0.90), reflecting lower HI for *APOE2/3* than *APOE3/3* and *APOE4* women (Additional file 1: Figure S1B). The difference for women was strengthened after adjustment for entorhinal cortex thickness (*F*(2,112) = 7.53,* p* < 0.001). However, sex by *APOE* interactions did not reach significance for cingulum ND (*p* = 0.28), entorhinal HI (*p* = 0.056), or any other measure. Exploratory analyses revealed an interaction between *APOE* and age for putamen RI (*p* = 0.003), with age-related decline present only for *APOE4* carriers (Fig. 2B).

### Interactions between APOE4 and cognitive status on brain microstructure

When models were conducted across the entire sample (both CN and CI) including cognitive status, *APOE4*, and their interaction, pronounced differences by cognitive status were observed, with additional differences by *APOE4*. As we previously reported in an overlapping sample [25], CI demonstrated widespread microstructural abnormalilties compared to CN (Additional file 1: Figure S2A), with the strongest differences in entorhinal cortex IF and HI, and hippocampal RI (*p* < 0.001); CI showed higher IF, and lower RI and HI, than CN. *APOE4* carriers exhibited lower entorhinal cortex HI and global gray matter ND and HI, as well as higher thalamic RI and ND, and global gray matter IF, than non-carriers (Additional file 1: Figure S2B).

**Fig. 3:**
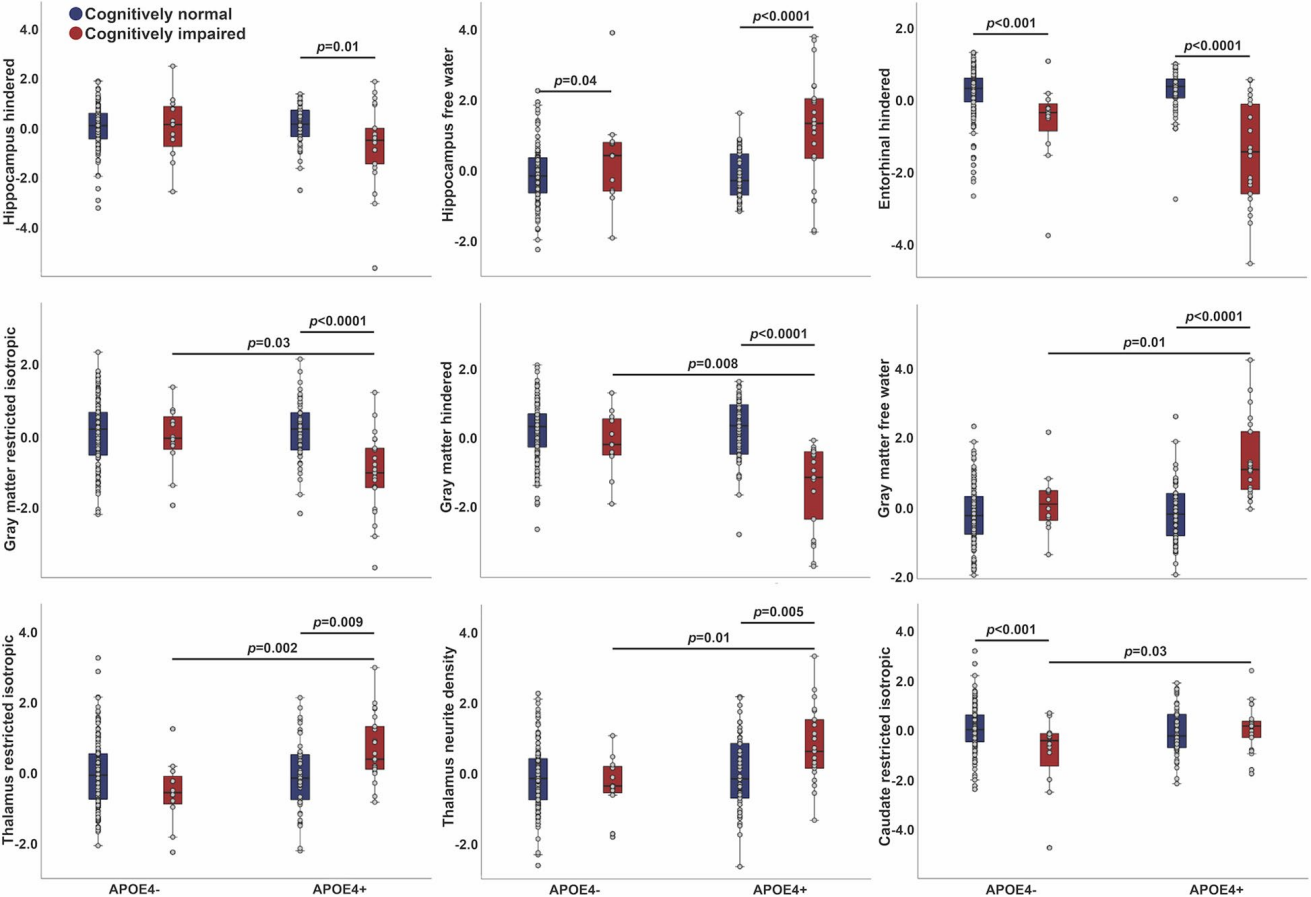
Interactions between *APOE4* and cognitive status on brain microstructure. RSI measures demonstrating significant interactions between *APOE4* and cognitive status are shown. Values are residuals, adjusted for age, sex, and scanner

*APOE4* interacted with cognitive status for hippocampal IF and HI (*p* < 0.05), and for entorhinal HI (*p* = 0.004) (Additional file 1: Table S8, Additional file 1: Figure S2C). As shown in Fig. 3, CI showed higher hippocampal IF and lower entorhinal cortex HI than CN among both *APOE4* carriers and non-carriers, with more pronounced differences for carriers. CI showed lower hippocampal HI than CN among *APOE4* carriers only. After adjustment for cortical thickness or volume respectively, the interactions for entorhinal cortex HI (*p* = 0.14) and hippocampal IF (*p* = 0.16) and HI (*p* = 0.17) were attenuated, driven by greater atrophy with cognitive impairment among *APOE4* carriers. Exploratory analyses also revealed significant interactions for global gray matter RI, HI, and IF (*p* < 0.01), thalamus RI (*p* < 0.001) and ND (*p* = 0.009), and caudate RI (*p* = 0.003), although whole-brain analyses revealed no signifcant localized interactions within cortical gray matter. Among *APOE4* carriers, CI exhibited lower gray matter RI and HI and higher IF, yet higher thalamic RI and ND, than CN. Among *APOE4* non-carriers, CI showed lower caudate RI than CN. Results were unchanged by further adjustment for diastolic blood pressure. Because education levels were higher among CI than CN (Additional file 1: Table S3), models were repeated with further adjustment for education, which did not alter results.

To evaluate region-specific associations among microstructural metrics, correlations among RSI measures within any region demonstrating significant effects of *APOE* are presented in Additional file 1: Table S9.

## Discussion

Leveraging the improved characterization of cellular microstructure by RSI, this study extends prior evidence of *APOE*-associated brain atrophy and diffusion abnormalities to further delineate modifying effects of *APOE4* at key stages along the cognitive aging spectrum, and to help illuminate the lesser understood role of *APOE2* in brain aging. Results revealed effects of *APOE4* on microstructure localized to the entorhinal cortex, which is among the earliest cortical targets of tau neuropathology, in contrast to effects of *APOE2* on cingulum microstructure. Sex stratification revealed that effects of *APOE2* and *APOE4* on brain microstructure were present only among women, extending prior evidence that *APOE* more profoundly modifies AD risk for women than men [16]. Findings implicate differential regulation of disease- and aging-related cytoarchitectural changes by *APOE* genotype, such that *APOE4* carriers undergo entorhinal neurodegeneration in preclinical and prodromal AD, with age-related striatal changes, whereas *APOE4* non-carriers present with age-related entorhinal cortex microstructural abnormalities and striatal injury accompanying cognitive impairment.

Among cognitively intact individuals, entorhinal neurite density was lower for *APOE4* carriers, which could reflect loss of dendritic density or complexity, or axonal damage occurring early along the AD trajectory. The regional specificity of these findings aligns with evidence that entorhinal cortex is a site of preclinical neuropathological tau deposition that is elevated for *APOE4* carriers [37] and predicts cognitive decline [38], and which undergoes accelerated atrophy in preclinical AD [39]. However, longitudinal examination is needed to distinguish preclinical neurodegeneration from pre-existing morphometric vulnerability that may lower brain reserve, as *APOE4* carriers demonstrate lower entorhinal cortex thickness even in youth [40]. In contrast, entorhinal RI declined with age among *APOE4* non-carriers, which may reflect aging-related neuronal loss or dystrophy among those with reduced genetic risk for AD, consistent with age-related entorhinal atrophy observed in the general population [41]. Given the challenge of distinguishing neurodegenerative from age-related structural brain changes, this dissociation of microstructural features affected by *APOE4* (ND) and aging (RI) within a region highlights the value of multicompartment diffusion MRI for developing more discriminative cytoarchitectural biomarkers. Notably, associations of entorhinal microstructural with *APOE* and age were independent of cortical thinning, pointing to more subtle preclinical cytoarchitectural changes among individuals with elevated AD risk that may precede atrophy. Because of our hypothesis-driven focus on the entorhinal cortex and hippocampus in a priori analyses, these analyses did not adjust for multiple comparisons across regions. However, whole-brain exploratory cortical surface-based analyses identified no significant differences by *APOE*, warranting caution when interpreting the regional specificity of the entorhinal cortex effects.

Cingulum ND was reduced for *APOE2* carriers relative to non-carriers, an unexpected observation indicating a lower fraction of oriented restricted diffusion in a major association fiber among those with low probability of converting to AD. WM ND is influenced by various tissue properties, which may be reduced with lower axonal density or myelination, which presents a significant barrier to water diffusion. Consistent with our finding, Westlye and colleagues [8] observed higher WM mean and radial diffusivity for *APOE2* carriers than *APOE3* homozygotes. However, others reported no difference in WM integrity for *APOE2* carriers [7, 15] and one DTI study identified higher fractional anisotropy for *APOE2* carriers than *APOE3* homozygotes within a small cluster of the posterior cingulum [14]. Further investigation is needed to reconcile these incongruent findings, which may be attributed to the inability of DTI to account for crossing fibers, the sample size of *APOE3* homozygotes in [14] or regional variability in WM organization within the cingulum. However, the functional implications of our finding warrant further investigation and may indicate WM vulnerability among *APOE2*-carriers, perhaps related to their heightened cerebrovascular risk. Alternatively, *APOE2*-related reductions in WM neurite density may reflect axonal organization supporting more efficient structural connectivity and restriction of neuropathological spread. Indeed, “large world” connectivity of distal networks including projections of the cingulate with frontal and temporal cortex [42] predominates in AD, accompanied by degraded “rich hubs,” localized networks of high connectivity [43]. Higher brain network segregation may also attenuate cortical tau propagation [44]. Thus, reduced structural connectivity of long-range association fibers such as the cingulum may optimize “small world” organization over distal connectivity, which may reinforce circuits supporting cognitive functions that decline with age.

Sex-stratified analyses suggested that observed effects of both *APOE2* and *APOE4* on entorhinal (ND and HI) and cingulum (ND) microstructure were limited to women, highlighting the importance of sex stratification in investigations of *APOE*. Women are more vulnerable to effects of *APOE4* on memory decline [45] as well as risk for AD dementia [16] and neuropathology [46], and more limited data suggest that *APOE2* also confers greater protection against AD risk for women than men [16]. Our findings extend this evidence to identify entorhinal and white microstructure as novel neurodegenerative markers of this sex disparity. Given that interactions between sex and *APOE* did not reach significance, further study is warranted to replicate our findings in larger datasets and to identify mechanisms by which sex-specific hormonal, sociocultural, or other factors interact with *APOE*.

Despite previous reports of WM compromise among *APOE4*-carriers [3–5], our data revealed no robust difference in WM microstructure by *APOE4* status. However, widespread WM differences by cognitive status were observed, consistent with our prior findings in an overlapping sample [25], that were not modified by *APOE4*. These findings would be consistent with a pathological cascade that originates with medial temporal neurodegeneration, followed by WM degeneration as neuropathology and associated cellular injury spreads throughout the cortex with disease progression. Further replication and longitudinal investigation are needed to clarify the temporal sequence of neurodegeneration and underlying mechanisms.

Our findings extend widely reported microstructural abnormalities in individuals with MCI [47] to demonstrate that *APOE4* modifies the microstructural signature of MCI, perhaps reflecting unique etiologies within this notably heterogeneous condition. While entorhinal hindered diffusion was lower and hippocampal free water was higher for cognitively impaired participants across the full sample, these differences were accentuated among *APOE4* carriers, consistent with their role as preclinical targets of AD neuropathology and neurodegeneration. These effects were partially attributable to atrophy with cognitive impairment, as has been widely reported previously, in contrast to the atrophy-independent effects of *APOE* on entorhinal microstructure observed among cognitively normal individuals. *APOE4* carriers also exhibited abnormal cortical gray matter and thalamic microstructure with MCI, patterns that were not present among non-carriers. Reduced gray matter RI and HI, which may represent cell death or dystrophy, and increased free water in cognitively impaired *APOE4* carriers are consistent with cortical atrophy that propagates throughout the neocortex with AD progression. The role of thalamic structure and function in AD appears complex and perhaps nonlinear, as thalamic atrophy in MCI [48] and hypometabolism in *APOE4* carriers [49] have been observed, whereas others have reported increased thalamic volume [50] and hypermetabolism with amyloid pathology [51] among *APOE4* carriers. Elevated thalamic RI and ND for cognitively impaired *APOE4* carriers could reflect morphological changes occurring with gliosis including somatic swelling of activated microglia, increased density and length of processes of activated astrocytes, or proliferation of either glial cell type, consistent with the interpretation that increased thalamic volume reflects inflammatory signaling involving microglial activation [50]. *APOE4* non-carriers demonstrated a distinct pattern of reduced striatal intracellular integrity (caudate RI) with cognitive impairment, which may point to AD-independent striatal contributions to clinical symptoms, as various conditions including Parkinson’s disease and psychiatric disorders are accompanied by striatal dysfunction [52, 53]. In contrast, age-related decline in striatal intracellular integrity (putamen RI) occurred only for *APOE4* carriers. Together, these findings support a model in which *APOE4* carriers are particularly susceptible to preclinical neurodegenerative changes in medial temporal regions that progress with cognitive decline, whereas among *APOE4* non-carriers, microstructural changes in these regions are more closely tied to aging than cognitive impairment. Conversely, striatal intracellular injury may be a marker of *APOE4*-independent cognitive impairment that demonstrates more benign age-related changes in *APOE4* carriers.

Diastolic blood pressure was elevated for male *APOE4* carriers relative to non-carriers, consistent with a documented susceptibility of *APOE4* carriers to cardiovascular risk factors [54], including higher diastolic blood pressure observed in some cohorts [55–57]. Concordantly, adjustment for diastolic blood pressure slightly attenuated the difference in entorhinal ND by *APOE4* in the sex-combined sample, but minimally affected this difference within women, who showed no difference in blood pressure by *APOE*, and did not alter any other *APOE4* effects. Though sex differences in vascular effects of *APOE* remain poorly understood, these preliminary findings suggest that associations of *APOE4* with blood pressure may be stronger in men, in contrast to more pronounced associations between *APOE* and microstructural injury in women, such that blood pressure does not meaningfully account for the observed effects of *APOE4*. Nevertheless, considering the still elusive role of *APOE*-dependent vascular contributions to AD, further research is needed to better characterize the extent to which vascular dysfunction mediates effects of *APOE4* on neurodegeneration, and how these pathways differ by sex.

A limitation of this study is lack of AD biomarker characterization on the full sample, precluding consideration of amyloid and tau in statistical analyses, which could aid in informing potential associations between AD neuropathology and *APOE*-dependent mechanisms underlying microstructural differences. Nevertheless, available biomarker data demonstrated more AD-like profiles (lower CSF amyloid and higher tau) for *APOE4* carriers and CI participants, supporting the interpretation that these individuals were more likely to be in preclinical AD stages. Furthermore, the cross-sectional nature of this study precludes tracking patterns of change or nonlinear dynamics of diffusion markers over time. Because the sample almost entirely comprised non-Hispanic White participants and we were unable to account for racial/ethnic or ancestry differences, findings may not generalize to non-white participants, considering effects of *APOE* are not uniform across racial groups. Given the small number of *APOE4* homozygotes and lack of *APOE2* homozygotes, further investigation is needed to probe allele dose effects. Finally, it is important to highlight that RSI identifies diffusion patterns consistent with distinct tissue compartments, but cannot directly image cell morphometry or inform physiological mechanisms underlying microstructural properties. As for all biophysical models, accuracy of diffusion MRI measures relies on the validity of assumptions regarding cell architecture, as well as selection of protocol parameters, which may complicate interpretability.

## Conclusions

Together, our findings indicate that microstructural signatures of *APOE* genotype emerge prior to cognitive impairment in patterns that may differ by sex, with entorhinal neurite loss as a potential preclinical AD biomarker, and WM microstructure associated with reduced AD risk. *APOE* genotype may shape regionally specific patterns of cellular changes occurring with aging and neurodegenerative disease, with effects of normal aging predominating in the absence of pathogenic drivers. Patterns of microstructural abnormalities associated with mild cognitive impairment differed according to *APOE4*, serving as potential differential indicators of underlying etiology.

### Supplementary Information

**Additional file 1: Table S1.** Description of restriction spectrum imaging metrics. **Table S2.** Participant characteristics (mean ± SD or *N*(%)) by *APOE4 *for the full sample and stratified by cognitive status. **Table S3.** Participant characteristics (mean ± SD or *N*(%)) by cognitive status*. ***Table S4.** Amyloid and tau measures (mean ± SD or *N*(%)) by *APOE4* genotype and cognitive status for the subset of participants who underwent lumbar puncture. **Table S5.** Participant characteristics (mean ± SD or *N*(%)) by cohort and cognitive status*. ***Table S6.** Effect sizes (*F*-values) and *p*-values for differences in RSI metrics within all regions examined, by *APOE4* among cognitively normal participants*. ***Table S7.** Effect sizes (*F*-values) and *p*-values for differences in RSI metrics within all regions examined, by *APOE (APOE2/3* vs *APOE3/3* vs *APOE4*-carrier) among cognitively normal participants*. ***Table S8.** Interaction between *APOE4 *and cognitive impairment on brain microstructure (mean ± SD, adjusted for age, sex, and scanner). **Table S9.** Correlations (Pearson’s *r*) between RSI metrics within selected regions of interest demonstrating significant microstructural differences by *APOE*. **Figure S1.** Sex-specific differences in brain microstructure by *APOE* in cognitively normal participants. Entorhinal cortex neurite density was lower for *APOE4* carriers than for non-carriers among women only (A). Entorhinal cortex hindered isotropic diffusion (B) and cingulum neurite density (C) were lower for *APOE2/3* than for *APOE3/3* and *APOE4* carriers among women only. Values are residuals, adjusted for age and scanner. Pairwise comparisons in B and C are Bonferroni corrected for multiple comparisons. **Figure S2.** Effects of cognitive status and *APOE4*, and their interaction, on brain microstructure. *F*-values are illustrated for main effects of cognitive status (A), *APOE4* (B), and their interaction (C) on RSI metrics in entorhinal cortex, subcortical regions of interest, fiber tracts of interest, and global gray and white matter. Effect sizes for main effects are plotted such that positive values indicate means for cognitively normal (CN) > cognitively impaired (CI) and *APOE4* non-carriers > carriers, whereas negative values indicate means for CI > CN and *APOE4* carriers > non-carriers. Effects reaching significance after correction for multiple comparisons are highlighted with a black border.

## Data Availability

The datasets used in this study are available from the corresponding author on reasonable request. Data for the RBS are available at: https://knit.ucsd.edu/ranchobernardostudy/.

## Abbreviations

Aβ: Beta-amyloid
AD: Alzheimer’s disease
ADRC: Alzheimer’s disease research center
APOE: Apolipoprotein E
BMI: Body mass index
CI: Cognitively impaired
CN: Cognitively normal
DTI: Diffusion tensor imaging
FDR: False discovery rate
HI: Hindered isotropic
IF: Isotropic free water
MCI: Mild cognitive impairment
ND: Neurite density
RBS: Rancho Bernardo Study
RI: Restricted isotropic
RSI: Restriction spectrum imaging
WM: White matter
