# Supplementary material for: Effects of APOE2 and APOE4 on brain microstructure in older adults: modification by age, sex, and cognitive status
Source: Alzheimers Res Ther. 2024 Jan 11;16:7. doi: 10.1186/s13195-023-01380-w (PMC10782616; doi:10.1186/s13195-023-01380-w)
Supplement: Supplementary file 1 — Additional file 1: Table S1. Description of restriction spectrum imaging metrics. Table S2. Participant characteristics (mean ± SD or N(%)) by APOE4 for the full sample and stratified by cognitive status. Table S3. Participant characteristics (mean ± SD or N(%)) by cognitive status. Table S4. Amyloid and tau measures (mean ± SD or N(%)) by APOE4 genotype and cognitive status for the subset of participants who underwent lumbar puncture. Table S5. Participant characteristics (mean ± SD or N(%)) by cohort and cognitive status. Table S6. Effect sizes (F-values) and p-values for differences in RSI metrics within all regions examined, by APOE4 among cognitively normal participants. Table S7. Effect sizes (F-values) and p-values for differences in RSI metrics within all regions examined, by APOE (APOE2/3 vs APOE3/3 vs APOE4-carrier) among cognitively normal participants. Table S8. Interaction between APOE4 and cognitive impairment on brain microstructure (mean ± SD, adjusted for age, sex, and scanner). Table S9. Correlations (Pearson’s r) between RSI metrics within selected regions of interest demonstrating significant microstructural differences by APOE. Figure S1. Sex-specific differences in brain microstructure by APOE in cognitively normal participants. Entorhinal cortex neurite density was lower for APOE4 carriers than for non-carriers among women only (A). Entorhinal cortex hindered isotropic diffusion (B) and cingulum neurite density (C) were lower for APOE2/3 than for APOE3/3 and APOE4 carriers among women only. Values are residuals, adjusted for age and scanner. Pairwise comparisons in B and C are Bonferroni corrected for multiple comparisons. Figure S2. Effects of cognitive status and APOE4, and their interaction, on brain microstructure. F-values are illustrated for main effects of cognitive status (A), APOE4 (B), and their interaction (C) on RSI metrics in entorhinal cortex, subcortical regions of interest, fiber tracts of interest, and global gray and white ma [file 13195_2023_1380_MOESM1_ESM.docx]

**Supplementary Data:**

**Effects of *APOE2* and *APOE4* on brain microstructure in older adults: Modification by age, sex, and cognitive status**

Emilie T. Reas, PhD ^a^*, Curtis Triebswetter ^a^, Sarah J. Banks, PhD ^a^, Linda K. McEvoy, PhD ^b,c^,

^a^ Department of Neurosciences, University of California, San Diego

^b^ Herbert Wertheim School of Public Health and Human Longevity Science, University of California, San Diego

^e^ Kaiser Permanente Washington Health Research Institute, Seattle, WA

**Supplementary Table 1**. Description of restriction spectrum imaging metrics.

|  | **Diffusion scale (rate)** | **Diffusion orientation** | **Potential corresponding cytoarchitecture** |
| --- | --- | --- | --- |
| Restricted isotropic (RI) | Fine (slow) | Isotropic | Cell bodies |
| Neurite density (ND) | Fine (slow) | Anisotropic, accounting for multiple orientations | Neurites (axons, dendrites), glial processes |
| Hindered isotropic (HI) | Intermediate | Isotropic | Extracellular space, large cell bodies |
| Isotropic free water (IF) | Coarse (fast) | Isotropic | Cerebrospinal fluid |

**Supplementary Table 2**. Participant characteristics (mean±SD or *N*(%)) by *APOE4* for the full sample and stratified by cognitive status.

|  | **All (*N*=225)** | | | **Cognitively Normal (*N*=192)** | | | **Cognitively Impaired (*N*=33)** | | |
| --- | --- | --- | --- | --- | --- | --- | --- | --- | --- |
|  | **APOE4-**  ***N*=151** | **APOE4+**  ***N*=74** | **Group difference** | **APOE4-**  ***N*=139** | **APOE4+**  ***N*=53** | **Group difference** | **APOE4-**  ***N*=12** | **APOE4+**  ***N*=21** | **Group difference** |
| Age (years) | 76.9±7.3 | 75.8±6.1 | *F*(1,223)=1.40, *p*=0.24 | 76.6±7.3 | 75.4±5.6 | *F*(1,190)=1.117, *p*=0.28 | 80.3±7.6 | 76.6±7.3 | *F*(1,31)=1.95, *p*=0.17 |
| Sex (women) | *N*=85 (56%) | *N*=44 (59%) | *X*^2^(1)=0.20, *p*=0.65 | *N*=82 (59%) | *N*=37 (70%) | *X*^2^(1)=1.91, *p*=0.17 | *N*=3 (27%) | *N*=7 (33%) | *X*^2^(1)=0.25, *p*=0.62 |
| Education (years) | 15.4±2.3 | 16.0±2.5 | *F*(1,223)=2.41, *p*=0.12 | 15.3±2.2 | 15.5±2.4 | *F*(1,109)=0.41, *p*=0.52 | 17.5±2.3 | 17.1±2.4 | *F*(1,31)=0.19, *p*=0.66 |
| Cognitively impaired | 12 (8%) | 21 (28%) | ***X*^2^(1)=16.75, *p*<0.001** | *NA* | *NA* | *NA* | *NA* | *NA* | *NA* |
| SBP | 125.2±15.9 | 129.3±17.9 | *F*(1,217)=2.85, *p*=0.09 | 125.4±16.3 | 130.0±18.8 | *F*(1,184)=2.66, *p*=0.10 | 123.3±11.3 | 127.6±15.7 | *F*(1,31)=0.70, *p*=0.41 |
| DBP | 71.4±9.5 | 75.0±9.3 | ***F*(1,217)=6.99, *p*=0.009** | 71.3±9.4 | 75.4±9.0 | ***F*(1,184)=6.88, *p*=0.009** | 71.9±11.0 | 74.0±10.1 | *F*(1,31)=0.33, *p*=0.57 |
| BMI | 25.8±4.2 | 25.0±3.6 | *F*(1,219)=1.83, *p*=0.18 | 25.7±4.0 | 25.1±3.7 | *F*(1,186)=0.77, *p*=0.38 | 26.4±6.0 | 25.1±3.3 | *F*(1,30)=0.71, *p*=0.41 |
| Diabetes | *N*=23 (15%) | *N*=9 (12%) | *X*^2^(1)=0.41, *p*=0.52 | *N*=20 (14%) | *N*=6 (11%) | *X*^2^(1)=0.33, *p*=0.57 | *N*=3 (25%) | *N*=3 (14%) | *X*^2^(1)=0.59, *p*=0.44 |
| E2E3 / E3E3 (*N*) * | 22 / 128 | 0 / 0 | *NA* | 22 / 117 | 0 / 0 | *NA* | 0 / 12 | 0 / 0 | *NA* |
| E3E4 / E4E4 (*N*) | 0 / 0 | 63 / 11 | *NA* | 0 / 0 | 51 / 2 | *NA* | 0 / 0 / 0 | 12 / 9 | *NA* |

Body mass index (BMI) is adjusted for sex. DBP, diastolic blood pressure; SBP, systolic blood pressure * One cognitively normal participant had inconclusive *APOE2/3* vs *APOE3/3* genotype.

**Supplementary Table 3**. Participant characteristics (mean±SD or *N*(%)) by cognitive status*.*

|  | **Cognitively normal**  ***N*=192** | **Cognitively impaired**  ***N*=33** | **Group difference** |
| --- | --- | --- | --- |
| Age (years) | 76.3±6.8 | 77.9±7.5 | *F*(1,223)=1.56, *p*=0.21 |
| Sex (women) | *N*=119 (62%) | *N*=10 (30%) | ***X*^2^(1)=11.55, *p*<0.001** |
| Education (years) | 15.3±2.2 | 17.3±2.4 | ***F*(1,223)=20.84, *p*<0.001** |
| SBP | 126.6±17.1 | 126.0±14.2 | *F*(1,217)=0.04, *p*=0.84 |
| DBP | 72.4±9.5 | 73.3±10.3 | *F*(1,217)=0.22, *p*=0.64 |
| BMI | 25.6±3.9 | 25.0±4.4 | *F*(1,219)=0.60, *p*=0.44 |
| Diabetes | *N*=26 (14%) | *N*=6 (18%) | *X*^2^(1)=0.48, *p*=0.49 |

Body mass index (BMI) is adjusted for sex. DBP, diastolic blood pressure; SBP, systolic blood pressure

**Supplementary Table 4**. Amyloid and tau measures (mean±SD or *N*(%)) by *APOE4* genotype and cognitive status for the subset of participants who underwent lumbar puncture.

|  | ***APOE4*-**  ***N*=37** | ***APOE4*+**  ***N*=33** | **Group difference** | **CN**  ***N*=48** | **CI**  ***N*=22** | **Group difference** |
| --- | --- | --- | --- | --- | --- | --- |
| AD pathology+ | *N*=15 (41%) | *N*=22 (67%) | ***X*^2^(1)=4.78, *p*=0.03** | *N*=18 (38%) | *N*=19 (86%) | ***X*^2^(1)=14.45, *p*<0.001** |
| t-tau/Aβ42 | 0.61±0.54 | 0.95±0.56 | ***F*(1,67)=6.44, *p*=0.01** | 0.59±0.47 | 1.17±0.61 | ***F*(1,67)=18.62, *p*<0.001** |
| Aβ42/40 | 0.078±0.027 | 0.054±0.021 | ***F*(1,67)=16.82, *p*<0.001** | 0.072±0.026 | 0.054±0.026 | ***F*(1,67)=7.48, *p*=0.008** |
| t-tau | 389±224 | 466±243 | *F*(1,67)=1.92, *p*=0.17 | 371±188 | 545±284 | ***F*(1,67)=9.38, *p*=0.003** |
| p-tau | 45.3±24.2 | 70.7±40.7 | ***F*(1,67)=10.29, *p*=0.002** | 48.1±26.4 | 77.4±43.6 | ***F*(1,67)=12.12, *p*<0.001** |

Biomarker levels are adjusted for time between lumbar puncture and MRI. t-tau/Aβ42>0.54 is considered positive for Alzheimer’s disease (AD) pathology. CN, cognitively normal; CI, cognitively impaired

**Supplementary Table 5**. Participant characteristics (mean±SD or *N*(%)) by cohort and cognitive status*.*

|  | **RBS**  **Cognitively normal**  ***N*=138** | **RBS**  **Cognitively impaired**  ***N*=1** | **ADRC**  **Cognitively normal**  ***N*=54** | **ADRC**  **Cognitively impaired**  ***N*=32** |
| --- | --- | --- | --- | --- |
| Age (years) | 76.5±6.4 | 80.9 | 75.8±5.2 | 77.8±7.6 |
| Sex (women) | *N*=85 (62%) | *N*=0 | *N*=34 (63%) | *N*=10 (31%) |
| Education (years) | 14.9±2.0 | 16 | 16.3±2.4 | 17.3±2.4 |
| MMSE (3MS) | 28.9±1.3 (95.1±3.7) | 22 (70) | 29.1±1.3 | 25.6±4.5 |
| SBP | 127.0±18.1 | 119.0 | 125.8±14.0 | 126.2±14.4 |
| DBP | 71.2±9.6 | 66.5 | 75.7±8.4 | 73.5±10.4 |
| BMI | 26.0±3.9 | 22.9 | 24.4±3.7 | 25.6±4.5 |
| Diabetes | *N*=25 (18%) | *N*=1 | *N*=1 (2%) | *N*=5 (16%) |
| E2E3 / E3E3 (*N*) * | 19 / 86 | 0 / 1 | 3 / 30 | 0 / 11 |
| E3E4 / E4E4 (*N*) | 31 / 1 | 0 / 0 | 20 / 1 | 12 / 9 |

* One cognitively normal RBS participant had inconclusive *APOE2/3* vs *APOE3/3* genotype.

Because the 3MS was administered for RBS participants, both 3MS and derived MMSE scores are reported.

Body mass index (BMI) is adjusted for sex. ADRC, UC San Diego Shiley-Marcos Alzheimer’s Disease Research Center; DBP, diastolic blood pressure; RBS, Rancho Bernardo Study; SBP, systolic blood pressure

**Supplementary Table 6**. Effect sizes (*F*-values) and *p*-values for differences in RSI metrics within all regions examined, by *APOE4* among cognitively normal participants*.*

|  | **Restricted isotropic** | | **Neurite density** | | **Isotropic free water** | | **Hindered isotropic** | |
| --- | --- | --- | --- | --- | --- | --- | --- | --- |
|  | ***F*-value** | ***p*-value** | ***F*-value** | ***p*-value** | ***F*-value** | ***p*-value** | ***F*-value** | ***p*-value** |
| Entorhinal | 0.39 | 0.53 | **4.46** | **0.04** | 0.01 | 0.92 | 0.94 | 0.33 |
| Hippocampus | 0.00 | 0.98 | 2.09 | 0.15 | 0.09 | 0.78 | 0.21 | 0.65 |
| Cingulum | 0.15 | 0.70 | 2.78 | 0.10 | 0.86 | 0.35 | *NA* | *NA* |
| Corpus callosum | 0.00 | 0.95 | 0.14 | 0.71 | 0.61 | 0.43 | *NA* | *NA* |
| Fornix | 1.63 | 0.20 | 0.48 | 0.49 | 2.52 | 0.11 | *NA* | *NA* |
| Inferior fronto-occipital | 0.00 | 0.97 | 0.47 | 0.49 | 0.44 | 0.51 | *NA* | *NA* |
| Inferior longitudinal | 0.72 | 0.40 | 1.30 | 0.26 | 0.59 | 0.44 | *NA* | *NA* |
| Parahippocampal cingulum | 0.15 | 0.70 | 0.01 | 0.93 | 1.54 | 0.22 | *NA* | *NA* |
| Superior longitudinal | 0.95 | 0.33 | 0.59 | 0.44 | 0.14 | 0.71 | *NA* | *NA* |
| Uncinate | 0.72 | 0.40 | 1.11 | 0.29 | 0.02 | 0.89 | *NA* | *NA* |
| Thalamus | 0.14 | 0.71 | 0.39 | 0.53 | 0.08 | 0.78 | 0.03 | 0.87 |
| Caudate | 1.02 | 0.31 | 0.16 | 0.69 | 0.37 | 0.54 | 0.01 | 0.94 |
| Putamen | 0.16 | 0.69 | 0.00 | 0.98 | 0.04 | 0.84 | 0.29 | 0.59 |
| Amygdala | 0.63 | 0.43 | 0.01 | 0.91 | 0.00 | 0.97 | 0.00 | 0.95 |
| Mean gray matter | 0.13 | 0.72 | 0.36 | 0.55 | 0.11 | 0.74 | 0.05 | 0.81 |
| Mean white matter | 0.12 | 0.73 | 1.15 | 0.28 | 0.00 | 0.95 | *NA* | *NA* |

**Supplementary Table 7**. Effect sizes (*F*-values) and *p*-values for differences in RSI metrics within all regions examined, by *APOE (APOE2/3* vs *APOE3/3* vs *APOE4*-carrier) among cognitively normal participants*.*

|  | **Restricted isotropic** | | **Neurite density** | | **Isotropic free water** | | **Hindered isotropic** | |
| --- | --- | --- | --- | --- | --- | --- | --- | --- |
|  | ***F*-value** | ***p*-value** | ***F*-value** | ***p*-value** | ***F*-value** | ***p*-value** | ***F*-value** | ***p*-value** |
| Entorhinal | 0.31 | 0.73 | 2.29 | 0.10 | 0.35 | 0.71 | 1.73 | 0.18 |
| Hippocampus | 0.50 | 0.61 | 1.01 | 0.37 | 0.83 | 0.44 | 2.71 | 0.07 |
| Cingulum | 0.07 | 0.93 | **5.42** | **0.005** | 1.01 | 0.36 | *NA* | *NA* |
| Corpus callosum | 0.02 | 0.98 | 0.86 | 0.42 | 0.52 | 0.60 | *NA* | *NA* |
| Fornix | 1.03 | 0.36 | 1.01 | 0.37 | 1.51 | 0.22 | *NA* | *NA* |
| Inferior fronto-occipital | 0.25 | 0.78 | 0.38 | 0.68 | 0.91 | 0.40 | *NA* | *NA* |
| Inferior longitudinal | 0.36 | 0.70 | 1.14 | 0.32 | 0.60 | 0.55 | *NA* | *NA* |
| Parahippocampal cingulum | 0.49 | 0.62 | 0.34 | 0.71 | 0.87 | 0.42 | *NA* | *NA* |
| Superior longitudinal | 0.47 | 0.63 | 1.68 | 0.19 | 0.08 | 0.92 | *NA* | *NA* |
| Uncinate | 0.53 | 0.59 | 1.18 | 0.31 | 0.72 | 0.49 | *NA* | *NA* |
| Thalamus | 0.93 | 0.40 | 0.30 | 0.74 | 0.35 | 0.71 | 1.33 | 0.27 |
| Caudate | 3.08 | 0.05 | 2.22 | 0.11 | 0.22 | 0.80 | 0.39 | 0.09 |
| Putamen | 2.46 | 0.09 | 1.52 | 0.22 | 0.06 | 0.94 | 2.04 | 0.13 |
| Amygdala | 0.63 | 0.53 | 0.15 | 0.86 | 0.05 | 0.95 | 0.93 | 0.40 |
| Mean gray matter | 0.24 | 0.78 | 0.31 | 0.73 | 0.98 | 0.38 | 1.30 | 0.27 |
| Mean white matter | 0.07 | 0.94 | 1.19 | 0.31 | 0.12 | 0.88 | *NA* | *NA* |

**Supplementary Table 8.** Interaction between *APOE4* and cognitive impairment on brain microstructure (mean±SD, adjusted for age, sex, and scanner).

|  | ***APOE4* non-carrier** | | ***APOE4* carrier** | |  |
| --- | --- | --- | --- | --- | --- |
| **RSI metric** | **CN *N*=139** | **CI *N*=12** | **CN *N*=53** | **CI *N*=21** | ***APOE4* x group** |
| Hippocampus HI | 0.76±0.04 | 0.75±0.07 | 0.76±0.04 | 0.73±0.08** | *F*(1,217)=4.05, *p*=0.046 |
| Hippocampus IF | 0.42±0.08 | 0.44±0.13** | 0.42±0.10 | 0.48±0.12** | *F*(1,217)=5.03, *p*=0.03 |
| Entorhinal HI | 0.78±0.09 | 0.69±0.11** | 0.78±0.08 | 0.60±0.15** | *F*(1,218)=8.25, *p*=0.004 |
| Mean gray matter RI | 0.28±0.03 | 0.27±0.04 | 0.28±0.03 | 0.25±0.04*,** | *F*(1,216)=7.70, *p*=0.006 |
| Mean gray matter HI | 0.72±0.05 | 0.70±0.07 | 0.72±0.05 | 0.66±0.07*,** | *F*(1,216)=12.55, *p*<0.001 |
| Mean gray matter IF | 0.46±0.09 | 0.48±0.11 | 0.46±0.10 | 0.53±0.10*,** | *F*(1,216)=10.23, *p*=0.002 |
| Thalamus RI | 0.38±0.04 | 0.37±0.06 | 0.38±0.04 | 0.41±0.04*,** | *F*(1,214)=11.15, *p*<0.001 |
| Thalamus ND | 0.44±0.03 | 0.43±0.03 | 0.44±0.03 | 0.47±0.03*,** | *F*(1,214)=6.91, *p*=0.009 |
| Caudate RI | 0.36±0.05 | 0.31±0.09** | 0.35±0.06 | 0.36±0.05* | *F*(1,216)=8.85, *p*=0.003 |

Regions with an *APOE4* x group interaction at *p*<0.05 Bonferroni corrected for multiple comparisons are shown. Raw *p*-values are reported. CI, cognitively impaired; CN, cognitively normal; HI, hindered isotropic; IF, isotropic free water; ND, neurite density; RI, restricted isotropic * *p*<0.05 versus *APOE4* non-carrier, ** *p*<0.05 versus cognitively normal

**Supplementary Table 9.** Correlations (Pearson’s *r*) between RSI metrics within selected regions of interest demonstrating significant microstructural differences by *APOE*.

| **Region** | **RI - ND** | **RI - IF** | **RI - HI** | **ND - IF** | **ND - HI** | **IF - HI** |
| --- | --- | --- | --- | --- | --- | --- |
| Entorhinal | r=0.47, p<0.001 | r=-0.71, p<0.001 | r=0.39, p<0.001 | r=-0.45, p<0.001 | r=0.25, p<0.001 | r=-0.85, p<0.001 |
| Mean gray matter | r=0.55, p<0.001 | r=-0.92, p<0.001 | r=0.79, p<0.001 | r=-0.46, p<0.001 | r=0.29, p<0.001 | r=-0.94, p<0.001 |
| Cingulum | r=0.25, p<0.001 | r=-0.72, p<0.001 | *NA* | r=-0.14, p=0.049 | *NA* | *NA* |
| Hippocampus | r=0.67, p<0.001 | r=-0.83, p<0.001 | r=0.48, p<0.001 | r=-0.54, p<0.001 | r=0.27, p<0.001 | r=-0.87, p<0.001 |
| Thalamus | r=0.49, p<0.001 | r=-0.64, p<0.001 | r=-0.28, p<0.001 | r=-0.15, p=0.04 | r=-0.59, p<0.001 | r=-0.49, p<0.001 |
| Caudate | r=0.50, p<0.001 | r=-0.75, p<0.001 | r=0.12, p=0.10 | r=-0.12, p=0.10 | r=-0.46, p<0.001 | r=-0.68, p<0.001 |
| Putamen | r=0.59, p<0.001 | r=-0.60, p<0.001 | r=-0.34, p<0.001 | r=-0.07, p=0.39 | r=-0.58, p<0.001 | r=-0.50, p<0.001 |

HI, hindered isotropic; IF, isotropic free water; ND, neurite density; RI, restricted isotropic

**
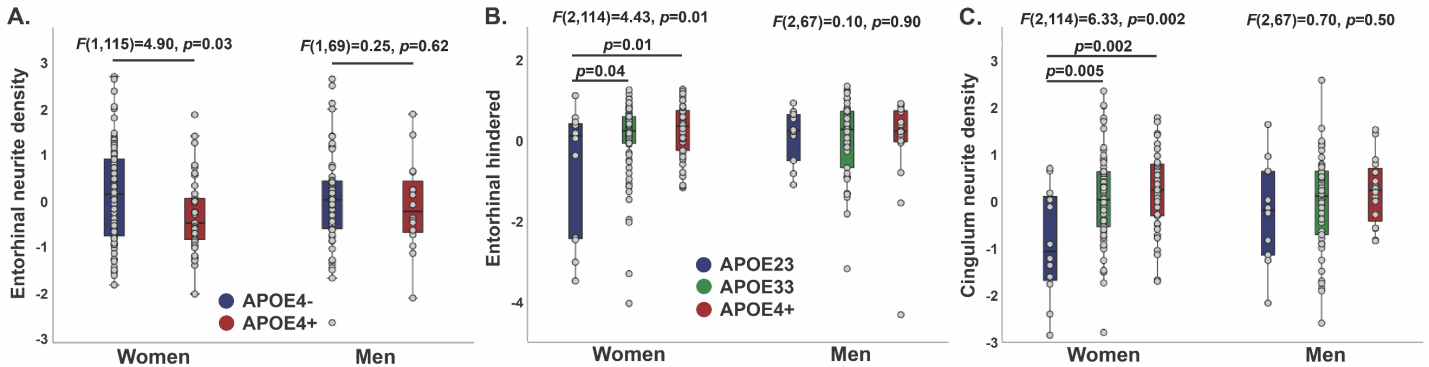
**

**Supplementary** **Figure 1**. **Sex-specific differences in brain microstructure by *APOE* in cognitively normal participants**. Entorhinal cortex neurite density was lower for *APOE4* carriers than for non-carriers among women only (A). Entorhinal cortex hindered isotropic diffusion (B) and cingulum neurite density (C) were lower for *APOE2/3* than for *APOE3/3* and *APOE4* carriers among women only. Values are residuals, adjusted for age and scanner. Pairwise comparisons in B and C are Bonferroni corrected for multiple comparisons.


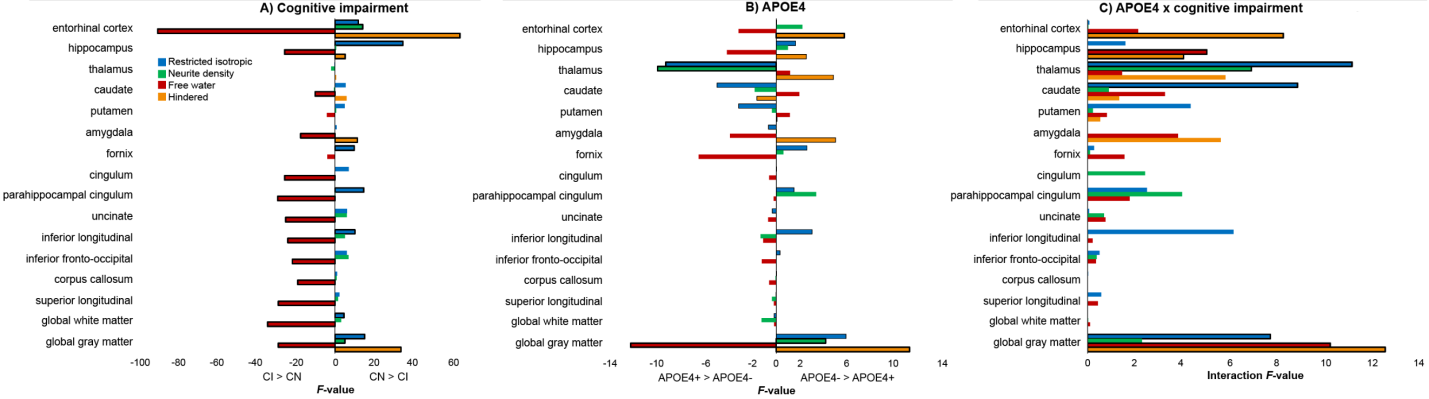


**Supplementary Figure 2. Effects of cognitive status and *APOE4*, and their interaction, on brain microstructure.** *F*-values are illustrated for main effects of cognitive status (A), *APOE4* (B), and their interaction (C) on RSI metrics in entorhinal cortex, subcortical regions of interest, fiber tracts of interest, and global gray and white matter. Effect sizes for main effects are plotted such that positive values indicate means for cognitively normal (CN) > cognitively impaired (CI) and *APOE4* non-carriers > carriers, whereas negative values indicate means for CI > CN and *APOE4* carriers > non-carriers. Effects reaching significance after correction for multiple comparisons are highlighted with a black border
